# Regulation of pancreatic cancer cell migration and invasion by RhoC GTPase and Caveolin-1

**DOI:** 10.1186/1476-4598-4-21

**Published:** 2005-06-21

**Authors:** Min Lin, Melinda M DiVito, Sofia D Merajver, Madanamohan Boyanapalli, Kenneth L van Golen

**Affiliations:** 1 Department of Internal Medicine, Division of Hematology/Oncology, The University of Michigan Comprehensive Cancer Center, Ann Arbor, Michigan 48109, USA; 2 Department of Cell and Molecular Physiology, The University of North Carolina, Chapel Hill, North Carolina 27599, USA; 3 Department of Neuroscience, The University of Michigan Medical School, Ann Arbor, Michigan 48109, USA

**Keywords:** Pancreatic cancer, RhoC GTPase, caveolin-1, cell migration, metastasis, MAPK

## Abstract

**Background:**

In the current study we investigated the role of caveolin-1 (cav-1) in pancreatic adenocarcinoma (PC) cell migration and invasion; initial steps in metastasis. Cav-1 is the major structural protein in caveolae; small Ω-shaped invaginations within the plasma membrane. Caveolae are involved in signal transduction, wherein cav-1 acts as a scaffolding protein to organize multiple molecular complexes regulating a variety of cellular events. Recent evidence suggests a role for cav-1 in promoting cancer cell migration, invasion and metastasis; however, the molecular mechanisms have not been described. The small monomeric GTPases are among several molecules which associate with cav-1. Classically, the Rho GTPases control actin cytoskeletal reorganization during cell migration and invasion. RhoC GTPase is overexpressed in aggressive cancers that metastasize and is the predominant GTPase in PC. Like several GTPases, RhoC contains a putative cav-1 binding motif.

**Results:**

Analysis of 10 PC cell lines revealed high levels of cav-1 expression in lines derived from primary tumors and low expression in those derived from metastases. Comparison of the BxPC-3 (derived from a primary tumor) and HPAF-II (derived from a metastasis) demonstrates a reciprocal relationship between cav-1 expression and p42/p44 Erk activation with PC cell migration, invasion, RhoC GTPase and p38 MAPK activation. Furthermore, inhibition of RhoC or p38 activity in HPAF-II cells leads to partial restoration of cav-1 expression.

**Conclusion:**

Cav-1 expression inhibits RhoC GTPase activation and subsequent activation of the p38 MAPK pathway in primary PC cells thus restricting migration and invasion. In contrast, loss of cav-1 expression leads to RhoC-mediated migration and invasion in metastatic PC cells.

## Background

Caveolin-1 (cav-1) is the major structural component of small Ω-shaped plasma membrane invaginations called caveolae [1]. Caveolae regulate plasma membrane signal transduction, with cav-1 acting as a scaffolding molecule to sequester and organize multi-molecular signaling complexes [2,3]. Many proteins which regulate multiple cellular activities such as growth and survival contain a putative cav-1 binding domain [2-4]. Recent evidence suggests a crucial role for cav-1 in regulating cellular migration and metastasis[5-7]. In a tumor progression model of breast cancer, loss of cav-1 corresponded to increased metastasis, while ectopic expression of cav-1 inhibited metastasis[8]. Furthermore, disruption of the *Cav-1 *gene in transgenic mice promotes mammary tumorigenesis and increased formation of metastases[9]. Conversely, in esophageal squamous cell carcinoma, lung adenocarcinoma, prostate, colon and clear cell renal cancers, high levels of cav-1 protein is associated with increased metastatic potential[10-12]. The molecular mechanism(s) of how cav-1 regulates tumor cell migration and metastasis has not been thoroughly explored.

Recent immunohistochemical studies have implicated increased cav-1 expression as a poor prognostic factor for pancreatic adenocarcinoma (PC) [13]. In the present study, we set out to determine whether cav-1 played a role in PC cell migration and invasion; initial steps in the metastatic cascade. Additionally, we attempted to identify the molecules regulated by cav-1 that are involved in PC cell migration and invasion.

Numerous molecules have been identified which interact with cav-1 [14-18]. Among these are the small monomeric GTPases Ras and RhoA [3,4]. The Ras Homology or Rho-subfamily of GTPases are typically involved in actin cytoskeleton rearrangement during cellular migration (reviewed in [19]). RhoC GTPase is a member of the Rho-subfamily that is associated with aggressive and highly metastatic tumors including PC [20-28].

Several studies have implicated RhoC as the predominant Rho GTPase in PC tumors and its expression is associated with metastasis and decreased survival (6 month versus 12 month for patients whose tumor expressed low or no RhoC) [29]. In a study aimed at characterizing genes involved in PC, laser capture microdissection (LCM) was used to compare normal pancreatic ductal cells with pancreatic cancers by cDNA microarray analysis [30]. RhoC was overexpressed and found in primary tumors that were locally invasive and in tumors from a variety of metastatic sites [30]. Another cDNA microarray study compared LCM isolated samples from 10 PC tumor specimens with 5 chronic pancreatitis and 5 normal pancreas specimens [31]. In PC tumors, RhoC was overexpressed 3- to 6-fold compared to normal and 2- to 4-fold compared with chronic pancreatitis (Craig Logsdon, personal communication) [31]. In each of these studies RhoA, Rac1 and Cdc42 were not significantly altered nor were they associated with aggressive or metastatic disease.

Similar to Ras, Rho GTPases are activated by a complex network of regulatory proteins and exists in the cell in an inactive GDP-bound and active GTP-bound state [32,33]. Unlike Ras, no activating mutations have been identified for Rho proteins in human cancers [32,33]. Therefore, increased Rho activity appears to be due to aberrant expression and/or dysregulation of regulatory proteins[32,33]. Like several Rho-subfamily members, RhoC contains a putative cav-1 binding domain. In the present study we demonstrate that cav-1 expression in PC cell lines regulates RhoC activation and cellular migration and invasion through the mitogen activated protein kinase (MAPK) pathway.

## Results

### Caveolin-1 Expression in Pancreatic Cancer Cell Lines

**Figure 1:**
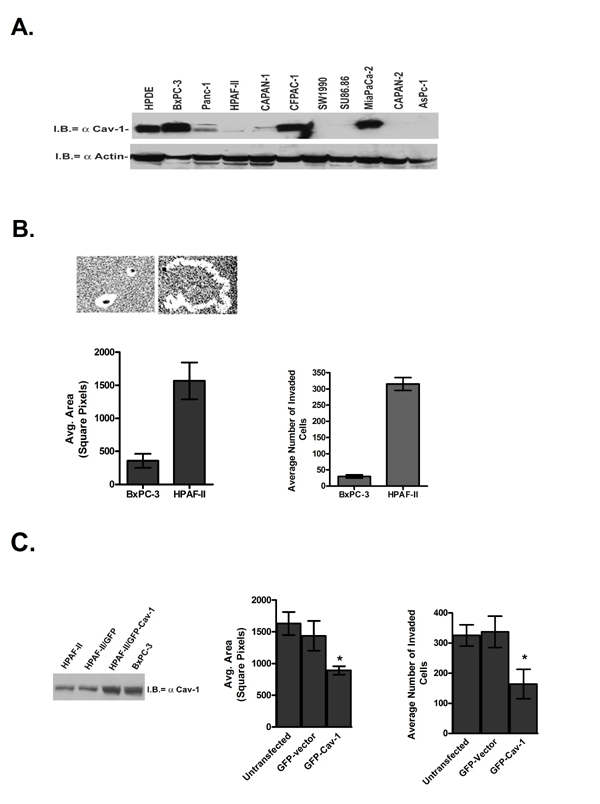
Caveolin-1 expression in human pancreatic adenocarcinoma cell lines. Panel A is a comparison of caveolin-1 protein expression in human ductal pancreatic epithelial (HPDE) and 10 pancreatic cancer cell lines. Aliquots of 50 μg total protein were probed by immunoblotting with a monoclonal antibody specific for caveolin-1. Both the α and β isoforms of caveolin-1 were detected. Immunoblot analysis of β-actin served as a loading control. B. Comparison of BxPC-3 and HPAF-II migration in a colloidal gold random migration assay. PC cells were seeded onto coverslips coated with colloidal gold, stimulated by the addition of 10% FBS and allowed to migrate for 16 h at 37°C. Phagokinetic tracks were photographed and areas measured. Invasion was measured by a Matrigel invasion assay using 10% serum as a chemoattractant. C. HPAF-II cells were transiently transfected with GFP-cav-1 or GFP only and their migratory capabilities measured in a blue-fluorescent bead migration assay in a manner identical to what was described for the colloidal gold assay. A representative Western blot demonstrating that ectopic cav-1 protein levels in the HPAF-II cells were similar to the BxPC-3 cell line. Phagokinetic tracks from GFP-expressing cells were imaged and areas measured. GFP-cav-1 cells were significantly less migratory (*p = 0.0032) and less invasive (*p = 0.002) than the controls.

Ten pancreatic adenocarcinoma (PC) cell lines were obtained from ATCC and compared with immortalized human pancreatic ductal epithelial (HPDE) cells for caveolin-1 (cav-1) expression. Figure 1A are the results of immunoblot analysis for total cellular cav-1 protein expression. Cav-1 expression was variable among the cell lines, with high cav-1 levels detected in the HPDE and PC cells derived from primary tumors (BxPC-3 and MiaPaCa-2). Comparatively, cav-1 levels in immortalized HPDE cells were lower than the BxPC-3 and MiaPaCa-2 cell lines. Expression was low or absent in cell lines derived from metastatic tumors or ascites fluid (HPAF-II, Capan-1, SW1990, SU86.86, Capan-2 and AsPc-1). The exception to this is the CFPAC-1 cell line, which was derived from a liver metastasis in a patient that had chronic cystic fibrosis. Interestingly, the Panc-1 cell line, which was derived from an invasive intraductal extension of a primary tumor, had an intermediate expression level. These results suggest that high cav-1 expression may be associated with primary tumors, while loss of cav-1 may be associated with metastases.

To determine whether cav-1 expression plays a central role in cell migration and invasion we chose the BxPC-3 and HPAF-II cell lines to represent tumor cells derived from primary tumors and metastases, respectively. Morphologically, both of the cell lines are similar and have a well-differentiated, epithelial appearance. We first tested the cells in a colloidal gold random motility assay to assess basal, non-ECM mediated migratory capabilities of cells. Figure 1B shows that after 16 h the BxPC-3 cell line was essentially non-migratory with an average phagokinetic track area of 357 ± 107 square pixels in contrast to the HPAF-II cell line which was highly migratory with an average track area of 1567 ± 227 square pixels. Similarly, when tested for their ability to invade through a Matrigel coated filter in response to a serum chemoattractant, the HPAF-II cells were 10-fold more invasive than the BxPC-3 cells.

Next we sought to establish whether loss of cav-1 was responsible for the increased migratory and invasive capabilities of the HPAF-II cells. To accomplish this we transiently transfected the HPAF-II cells with a GFP-tagged cav-1 expression vector. Using a variation of the colloidal gold assay, we compared untransfected, GFP-vector control and GFP-cav-1 transfected HPAF-II cells in a blue fluorescent bead random migration assay. This assay is performed the same as the colloidal gold assay, but allows us to fluorescently identify GFP-transfected cells. Figure 1C demonstrates that ectopic re-expression of cav-1 to levels similar to that of the BxPC-3 cells significantly decreases HPAF-II migration (890 ± 67 square pixels; p = 0.0032) compared with the untransfected or GFP-vector control HPAF-II cells (1630 ± 183 and 1435 ± 235 square pixels, respectively).

Similarly in a Matrigel invasion assay, re-expression of cav-1 in the HPAF-II cells decreased the invasiveness of the cells nearly 2-fold compared with the controls. The results from the motility and invasion experiments suggest that HPAF-II migration and invasion is mediated by cav-1.

### RhoC GTPase Induces PC Cell Migration

**Figure 2:**
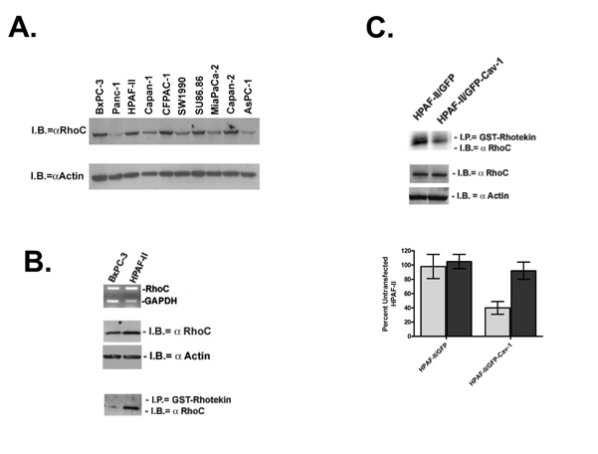
RhoC GTPase expression in BxPC-3 and HPAF-II cell lines. A. Total GDP/GTP-bound RhoC was measured in a panel of 10 PC cell lines. Protein (30 μg) was harvested from actively growing PC cell lines. B. Expression of RhoC in BXPC-3 and HPAF-II cells was measured by RT-PCR and immunoblot analysis using RhoC-specific primers and antibody, respectively. To determine the amount of active, GTP-bound RhoC a Rho activation assay was performed. GST-rhotekin is used to pull down RhoC-GTP from total cell lysates followed by immunoblotting with a RhoC-specific antibody. C. The RhoC activation assay was performed on the GFP-cav-1 HPAF-II transfectants 24 h after transfection. Differences in active and total RhoC levels were measured by densitometry and are represented.

Due to their role in cellular migration, invasion and metastasis the Rho GTPases were logical molecular candidates to interact with cav-1 to mediate cell migration and invasion. RhoC GTPase is prevalent in metastatic tumors, particularly in PC [20-28]. Hence, we chose to study RhoC GTPase in relationship to cav-1. As shown in Figure 2A RhoC GTPase is expressed on the protein level to varying degrees in the panel of 10 pancreatic cancer cell lines that were analyzed for cav-1 expression. However, more important than expression is the activation state of the GTPase. Figure 2B is a comparison of RhoC expression and activation in the BxPC-3 and HPAF-II cell lines. RT-PCR and immunoblot analysis confirm that RhoC is highly expressed on the mRNA and protein levels both in the BxPC-3 and HPAF-II cells. To determine the relative levels of active RhoC in these cell lines, a GST fusion protein of the Rho-binding domain of the downstream Rho effector protein, rhotekin, was used to selectively pull out GTP-bound RhoC. Although levels of total GDP/GTP-bound RhoC were similar for both cell lines, levels of active RhoC was considerably higher in the HPAF-II cell line.

To determine if cav-1 expression affected RhoC activation, levels of GTP-bound RhoC was measured in the GFP-cav-1 and control HPAF-II cells (Figure 2C). Compared with the controls, transient ectopic re-expression of cav-1 decreased levels of active RhoC by 6-fold without effecting total RhoC protein levels, suggesting regulation of RhoC activation by cav-1.

**Figure 3:**
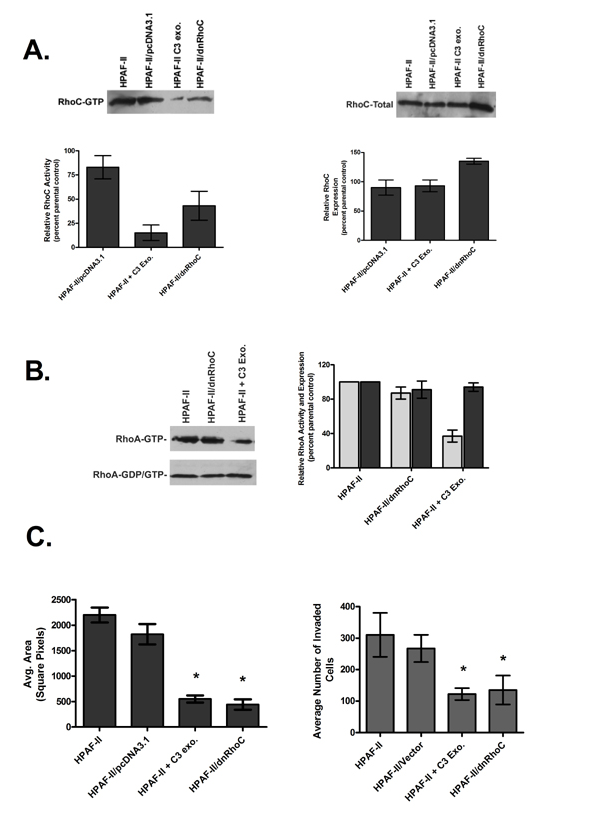
Establishment of stable, dominant negative RhoC HPAF-II transfectants. A. Results of a RhoC activation assay and total RhoC Western blot comparing wildtype, C3 exotransferase treated, vector control and dominant negative RhoC (dnRhoC) transfected HPAF-II cells. B. Comparison of total (dark grey) and active (light grey) RhoA levels utilizing a RhoA-specific antibody. C. Results of the effect of dnRhoC transfection and C3 treatment on HPAF-II cells in a colloidal gold migration assay. Inhibition of RhoC lead to a significant decrease in migration (*p = 0.001) and invasion (*p = 0.023).

Next, to directly implicate RhoC in PC cell migration and invasion, we generated stable HPAF-II dominant negative RhoC (dnRhoC) transfectants. For clarity and simplicity the results shown are from a polyclonal population which is representative of three individual clones that were tested. Figure 3A shows a 57% decrease of active RhoC in the HPAF-II/dnRhoC transfectants compared with the untransfected and vector transfected controls. As a positive control the HPAF-II cells were treated with C3 exotransferase. C3 exotransferase is a toxin derived from *Clostridium botulinum *and is effective at inhibiting RhoA, -B and -C activity with virtually no effect on Rac1 or Cdc42 [34,35]. C3 exotransferase treatment reduced active RhoC levels by 85%. As shown, both C3 exotransferase treatment and expression of dnRhoC did not significantly alter total levels of RhoC protein.

Dominant negative Rho GTPases work by entering into a non-productive interaction with Rho Guanine Exchange Factors (RhoGEFs), the proteins which catalyze the exchange of GDP for GTP. Due to the close homology between RhoC and RhoA (91% on the protein level), the possibility exists that both GTPases can be activated by the same RhoGEFs *in vivo*. As shown in Figure 3B, RhoA activity was not significantly altered by expression of dnRhoC. Again, as a positive control HPAF-II cells were treated with C3 exotransferase; this reduced RhoA activity an average of 63%. Therefore, RhoC activity was specifically decreased in the HPAF-II cells expressing dominant negative RhoC.

As shown in Figure 3C, inhibition of RhoC activity, either by dnRhoC or by treatment with C3 exotransferase, significantly reduced HPAF-II cell migration by nearly 4-fold (p = 0.001) and invasion by 3-fold (p = 0.023), suggesting a key role for RhoC GTPase mediating HPAF-II cell migration and invasion.

### Association of Cav-1 and RhoC GTPase in PC cells

Next, we considered the possibility that RhoC activity and PC migration and invasion are regulated by cav-1 through physical interaction of the GTPase with the scaffolding protein. Proteins that associate with cav-1 contain the canonical cav-1 binding domain, ΦXΦXXXXΦ or ΦXXXXΦXXΦ (where Φ= Trp, Phe or Tyr) [36,37]. A review of the RhoC protein sequence revealed a putative cav-1 binding sequence at amino acid residues 35–43 (YVPTVFENY).

**Figure 4:**
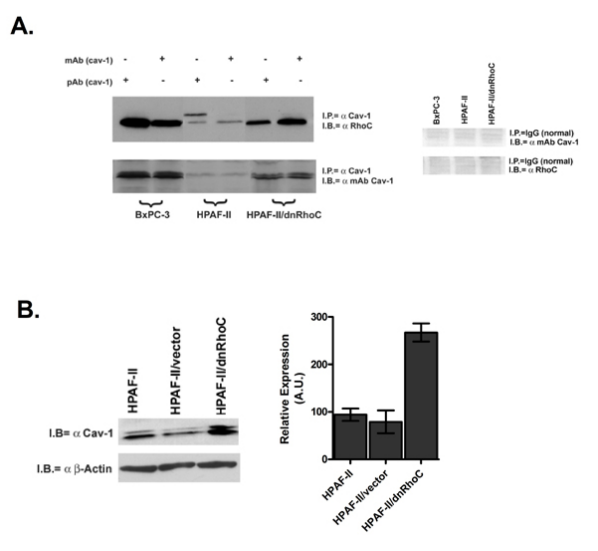
Interaction of cav-1 and RhoC in BxPC-3 and HPAF-II PC cell lines. A. Aliquots of 500 μg total protein from BxPC-3, HPAF-II and HPAF-II/dnRhoC transfectants were immunoprecipitated with either a monoclonal or polyclonal antibody to cav-1. Proteins were separated by SDS-PAGE, transferred to nitrocellulose and probed with either a RhoC-specific or cav-1 antibody. Normal IgG negative controls are also shown. B. Immunoblot analysis of 50 μg of total protein for total cav-1 expression using a cav-1 specific monoclonal antibody and actin was measured as a loading control. Densitometry was performed on each blot and expression levels were normalized to the actin control. Expression levels are represented as arbitrary units (AU).

Figure 4A are the results of immunoprecipitation assays using either a polyclonal or monoclonal antibody specific for cav-1 followed by immunoblotting for RhoC. Cav-1 and RhoC proteins co-immunoprecipitated in the BxPC-3 cells but not in the HPAF-II cell line. Unexpectedly, the association between cav-1 and RhoC was restored in the HPAF-II/dnRhoC cell line suggesting that inhibition of RhoC activity leads to re-expression of cav-1 protein.

The results of a cav-1 immunoblot for total cellular protein are shown in Figure 4B. Expression of cav-1 was increased 3.1-fold in the HPAF-II/dnRhoC cells compared with the parental HPAF-II and vector control cells. Actin was used as a loading control. Together with the immunoprecipitation data, these data suggest that cav-1 expression is in a reciprocal relationship with RhoC activation. High cav-1 protein expression leads to inhibition of RhoC activation while inhibition of RhoC activity leads to partial re-expression of cav-1. Furthermore, interaction of cav-1 and RhoC may result in decreased RhoC activation, limiting cell migration and invasion.

### PC Cell Migration Involves the Mitogen Activated Protein Kinase (MAPK) Pathway

Previous studies demonstrated that activation of p42/p44 extracellular regulated kinase (Erk) decreased cav-1 protein levels in constitutively-active Ras transformed NIH3T3 cells [38]. In the same set of studies it was shown that inhibition of oncogenic Ras-induced Erk activation with PD98059 increased cav-1 expression 5-fold [38]. That same group also demonstrated that ectopic re-expression of cav-1 decreased Erk activation in Ras-transformed CHO cells [39]. Our laboratory has previously demonstrated that RhoC can mediate inflammatory breast cancer cell migration and invasion through co-activation of the p42/p44 Erk and p38 arms of the MAPK pathway [40]. With these studies in mind we next examined whether RhoC can activate p42/p44 Erk in PC, subsequently decreasing cav-1 expression and leading to increased cellular migration. Also, we examined the potential involvement of p38 MAPK in this process.

**Figure 5:**
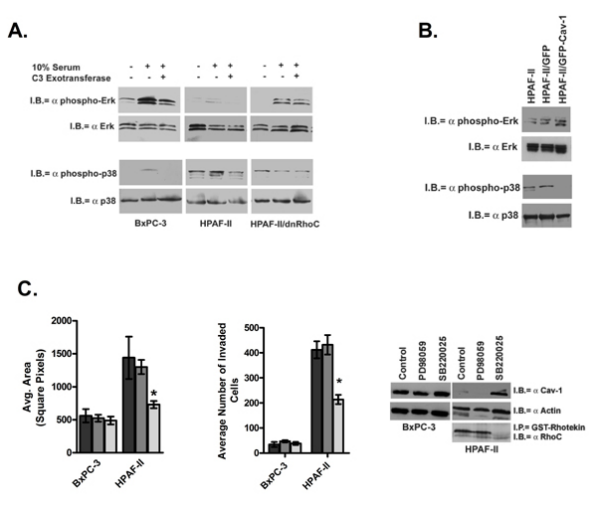
Analysis of the mitogen activated protein kinase pathway (MAPK) in the BxPC-3 and HPAF-II cells. A. Actively growing PC cells were serum starved for 24 h and left untreated or pretreated for 1 h with 5 μg C3 exotransferase, stimulated by the addition of 10% serum and proteins harvested 15 min later. Activated p42/p44 Erk and p38 MAPK were detected by antibodies specific for the phosphorylated forms of each of those proteins. Immunoblots were stripped and reprobed with antibodies specific for total forms of each protein. B. Comparison of active p42/p44 Erk and p38 MAPK in GFP-cav-1 and GFP-vector transient transfectants at 24 h after transfection. C. Results of a colloidal gold migration assay after 30 min pre-treatment of cells with 30 μM of either PD98059 or SB220025. Phagokinetic tracks were imaged at 6 h after stimulation. Results are given for DMSO control (dark grey), PD98059 treated (medium grey) and SB220025 treated (light grey). SB220025 treatment of the HPAF-II cells led to a significant decrease (*p = 0.001) in migration and invasion. Levels of cav-1 protein were increased, while active RhoC was decreased in SB220025 treated HPAF-II cells.

Figure 5A is a comparison of active (phospho-) and total levels of p42/p44 Erk and p38 MAPK in BxPC-3, HPAF-II and HPAF-II/dnRhoC cells that were serum starved for 16 h and stimulated with 10% serum alone or after pre-treatment with C3 exotransferase. Although active phospho-Erk levels were stimulated in both cell lines, active p42/p44 Erk was considerably higher in the BxPC-3 cell line compared with the HPAF-II cell line. Pretreatment with C3 exotransferase slightly decreased active Erk in serum stimulated cell lines. Similar results were previously demonstrated and are unexpected since the HPAF-II cell line harbors an activating G12D K-Ras mutation, while the BxPC-3 cell line has a wildtype K-Ras [41,42]. In the HPAF-II/dnRhoC cells, the overall phospho-p42/p44 levels were higher compared with the parental HPAF-II cell line suggesting that inhibition of RhoC leads to increased Erk activation.

Converse to what was observed for p42/p44 Erk the levels of phospho-p38 MAPK were low in the BxPC-3 and HPAF-II/dnRhoC cells and higher in the HPAF-II cells. C3 treatment decreased p38 activity in the BxPC-3 and HPAF-II cell lines. The levels of active p38 in the HPAF-II/dnRhoC and C3 treated HPAF-II cells were comparable; demonstrating an approximate 52% decrease in activity compared with the serum stimulated HPAF-II cells. Taken together, an inverse relationship between p42/p44 Erk and p38 MAPK signaling is suggested in the PC cells.

Levels of active Erk and p38 MAPK were assessed in the HPAF-II/GFP-cav-1 transfectants. As shown in Figure 5B, levels of active phospho-p42/p44 Erk increased in the GFP-cav-1 transfectants compared with the untransfected and control GFP-vector transfectants. Conversely, levels of phosphorylated p38 decreased in the cav-1 transfectants, mirroring what is observed in the HPAF-II/dnRhoC cells.

To tease out the individual roles of the p42/p44 Erk and p38 MAPK pathways in PC cellular migration, the BxPC-3 and HPAF-II cells were treated with the pharmacologic inhibitors PD98059 (to inhibit MEK1 and subsequently Erk) or SB220025 (to inhibit p38) and tested in migration and invasion assays (Figure 5C). SB220025 treatment significantly reduced HPAF-II migration and invasion (p = 0.001). PD98059 treatment had no effect on the cells ability to move in either assay suggesting that migration and invasion occurs through signaling of the p38 MAPK pathway.

Changes in cav-1 expression due to inhibitor treatment are also shown in Figure 5C. Cav-1 expression was slightly less after PD98059 treatment in both PC cell lines. Treatment with SB220025 increased cav-1 expression dramatically in the HPAF-II and slightly in the BxPC-3 cells, implying a reciprocal relationship between cav-1 expression and p38 activation. Furthermore, RhoC activity was appreciably reduced in the SB220025 treated HPAF-II cells strengthening the notion that cav-1 is in a reciprocal relationship with RhoC and p38 activity.

### Methyl-β-cyclodextrin increases PC cell motility

**Figure 6:**
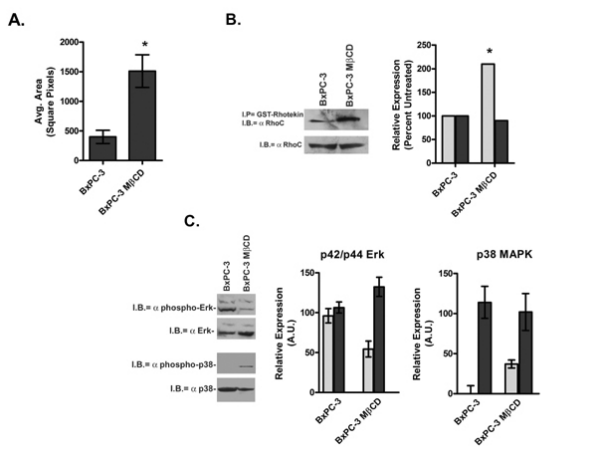
Effect of disruption of caveolae and mis-localization of caveolin-1 after treatment of BxPC-3 cells with MβCD. A. Results of a colloidal gold random motility assay performed after a 1 h pre-treatment of BxPC-3 cells with 5 mM MβCD in growth medium. Phagokinetic tracks were measured 16 h after stimulation. MβCD treated BxPC-3 cells were significantly more migratory than untreated cells (* p = 0.0001) B. Total (light grey) and active GTP-bound (dark grey) levels of RhoC were measured in MβCD treated BxPC-3 cells 3 h after treatment. A statistical difference in RhoC activation was reached with a p value of 0.0001. C. Immunoblot analysis of basal levels of active (light grey) and total (dark grey) p42/p44 Erk and p38 MAPK in MβCD treated BxPC-3 cells 3 h after treatment.

Lastly, we treated the BxPC-3 cell line with methyl-β-cyclodextrin (MβCD), which sequesters cholesterol, disrupts caveolae and mis-localizes cav-1 in the cell [43]. As shown in Figure 6A, MβCD treatment significantly increased the area of BxPC-3 migration (p = 0.0001). Increased cellular migration was accompanied by a significant increase in active GTP-bound RhoC (Figure 6B; p = 0.0001). Consistent with previous observations, Figure 6C demonstrates that levels of phospho-p42/p44 Erk in the MβCD treated cells decreased while levels of phospho-p38 MAPK increased. The changes in MAPK proteins approached but did not achieve statistical significance. These data further suggest that activation of RhoC GTPase is regulated by cav-1 and that RhoC signals through the p38 arm of the MAPK pathway to induce cellular migration.

## Discussion

Both loss and overexpression cav-1 has been described in tumor progression, sometimes within the same tumor type [44-47]. Loss and overexpression of cav-1 has also been associated with the progression to a metastatic phenotype within different cancers [47,8]. However, the molecular mechanism(s) by which cav-1 confers metastatic ability has not been thoroughly explored. Here we present evidence for the involvement of RhoC GTPase and p38 MAPK in the migratory and invasive phenotype after loss of cav-1 expression in PC cells.

Immunohistochemical staining of primary human PC tumors demonstrated that high cav-1 expression correlated with large PC tumor size and a poor prognosis [13]. In our current study we found that PC cell lines derived from primary tumors have high cav-1 expression while those derived from distant metastases have significantly reduced or lost expression. Two recent studies have demonstrated a biphasic expression of cav-1 in primary vs. metastatic tumors [48,49]. Both in renal cell and oral carcinomas, cav-1 is overexpressed in primary tumors but low or absent in distant metastases. It is possible that biphasic expression of cav-1; meaning, overexpression in primary tumors and low or absent expression in metastases occurs in pancreatic cancer.

Many proteins including the majority of the Rho GTPases have the ability to associate with cav-1 [3,4,14-18]. RhoC has been shown to be the predominant Rho protein in PC and is associated with particularly aggressive disease [21-29]. Given the role of RhoC in cellular migration and its prevalence in metastatic tumors, it was a logical choice to examine in relationship to cav-1.

One effect that high cav-1 expression could have in PC cells is regulating RhoC activity, thus limiting cell migration and promoting growth. This could potentially explain why PC cells harboring activating K-Ras mutations have lower activated phospho-p42/p44 Erk levels than expected [41,42]. Our data suggest that expression of high cav-1 levels in primary PC tumor cells favor the p42/p44 Erk pathway which is associated with cell growth and survival (reviewed in [50]). When cav-1 expression is diminished or lost, the p38 MAPK pathway is favored and cell migration occurs. Data in the current study also indicate that the p38 MAPK pathway leads to RhoC-mediated PC cell migration and invasion, while the Erk pathway does not. This is contrary to what we had demonstrated for RhoC-mediated migration and invasion in inflammatory breast cancer [40].

Experiments in NIH3T3 and CHO cells suggest a reciprocal and reversible relationship between p42/p44 Erk activation and cav-1 expression [38,39]. Our data suggests that this is not the case in PC cells. High cav-1 expression is associated with higher levels of active p42/p44 irregardless of K-Ras mutational status. The same studies also suggested a role for active protein kinase A (PKA) in reversibly suppressing cav-1 expression [38,39]. Our early studies suggested that this is also not the case in PC cells (data not shown). Instead our data suggest that suppression of RhoC or p38 MAPK activity restores cav-1 expression and suppresses migration and invasion.

This study is the first to draw a functional link between RhoC and cav-1 and to demonstrate involvement of the MAPK signal transduction pathway. This study also presents a fresh link to the regulation of RhoC-mediated migration and invasion, giving a functional role for RhoC in PC tumor cells.

## Conclusion

This study suggests that cav-1 expression suppresses RhoC GTPase activation and RhoC-mediated cellular migration and invasion. Further, expression of cav-1 favors activation of the p42/p44 Erk pathway, perhaps promoting growth and survival. Loss of cav-1 expression leads to increased RhoC activation favoring the p38 MAPK pathway and cellular migration and invasion. Inhibition of RhoC or p38 activation results in decreased cellular movement and partial re-expression cav-1 protein. These results may imply a biphasic expression of cav-1 during PC tumor progression promoting growth and survival in the primary tumor cells and motility in the metastatic cells. Lastly, this study suggests a functional role for RhoC GTPase in PC migration and invasion.

## Methods

### Cell Culture

All pancreatic cancer (PC) cell lines were obtained from American Type Culture Collection (ATCC; Rockville, MD) and grown in their required growth medium per ATCC description. Specifically, BxPC-3 cells were grown in 90% RPMI 1640 (Bio Whittaker, Walkersville, MD), 10% FBS (Biosource International, Rockville, MD) and HPAF-II cells in 90% EMEM (Bio Whittaker), 10% FBS. Human pancreatic ductal epithelial (HPDE) cells (a gift from Dr. Ming Sound Tsao) were grown in keratinocyte serum-free (KSF) medium with 0.2 ng/ml EGF and 30 μg/ml bovine pituitary extract (InVitrogen Gibco, Carlsbad, CA) [51,52]. In the inhibitor studies, cells were pre-incubated for 0.5 h with 30 μM PD98059 or SB220025, (Calbiochem, San Diego, CA) or as a control, with DMSO carrier and stimulated with normal growth medium. C3 exotransferase (5 μg) was introduced into cells as previously described using a lipid transfer mediated method [53,40] and treated for 1 h before analysis. BxPC-3 cells were treated with 5 mM MβCD in medium for 2 h.

### Plasmids and Transfection

Wild type human cav-1 was cloned from immortalized HPDE cells by RT-PCR. Total RNA was isolated from HPDE cells by Trizol (InVitrogen) and cDNA synthesized with the Wizard AMV-reverse transcriptase kit (Promega, Madison, WI). A 10 μl aliquot of cDNA was amplified by PCR, ligated into the pGEM-T Easy vector (Promega), and sequenced by the University of Michigan DNA sequencing core. The cav-1 sequence was confirmed using the NCBI BLAST database. The internal stop codon was removed using QuikChange (Stratagene, La Jolla, CA) and the insert transferred to pcDNA3.1/CT-GFP (InVitrogen). Cav-1 constructs were transiently transfected into PC cells using FuGene6 (Roche, Indianapolis, IN) and assayed 24 h later. Empty vector was used as a transfection controls. Dominant negative RhoC (dnRhoC) in pcDNA3.1 was obtained from Guthrie cDNA resource center. The dnRhoC construct or vector control was introduced into HPAF-II cells using FuGene6 and stable transfectants established by growing cells continuously in 250 μg/ml neomycin. Individual clones as well as a polyclonal population were chosen and tested in these experiments. Expression of the transgene was confirmed by RT-PCR for sequences unique to the plasmid. The results of the polyclonal population are reported because they are representative of the data obtained from all the clones.

### RT-PCR Analysis

RT-PCR of RhoC expression in PC cell lines was performed as previously described [54] using primers specific for RhoC GTPase and GAPDH control. Triplicate analysis of RT-PCR was performed.

### Immunoprecipitation/Western Blot Analysis

Protein was harvested from cells using RIPA buffer as previously described [54]. Lysates were separated by SDS-PAGE on a 4–15% gel, transferred to nitrocellulose, blocked and probed with a polyclonal antibody for RhoC [55], monoclonal antibodies for cav-1, (Pharmingen, San Diego, CA), RhoA (Cytoskelton Inc., Denver, CO.) or total and phospho- p42/p44 MAPK and p38 MAPK (Cell Signaling Technologies, Beverly, MA). After incubation with a goat anti-rabbit-HRP or goat anti-mouse-HRP antibody (Santa Cruz Biotechnology, Santa Cruz, CA), immunoblots were developed with Lumiglo (Cell Signaling Technologies), exposed to Hyperfilm (Amersham, Piscataway, NJ) and images recorded on an Alpha Image 950 documentation system (Alpha Innotech, San Leandro, CA). Densitometry was performed using ImageJ software (version 1.30; available from the NIH at ). Immunoprecipitation experiments were preformed by pre-incubating 1 mg of protein lysate with normal isotype control for 1 h followed by an overnight incubation with a polyclonal or monoclonal antibody for cav-1 (Pharmingen) and Protein A/G agarose PLUS (Santa Cruz) at 4°C. Precipitates were repeatedly pelleted by brief centrifugation followed by 4 washings with ice-cold PBS. Proteins were separated by SDS-PAGE on a 15% gel, transferred to nitrocellulose, blocked and probed with a polyclonal antibody to RhoC and exposed as described above.

### Rho Activation Assay

Rho activation assay reagents were supplied to us by John Collard, Ph.D. at the Netherlands Cancer Institute [56,57] and used to determine activation of RhoC in PC cells. Cells, 5–10 × 10^6 ^cells/assay, were grown in fresh serum-containing medium for 16 h and lysed with ice-cold GST-FISH buffer (10% Glycerol, 50 mM Tris pH 7.4, 100 mM NaCl, 1% NP-40 and 2 mM MgCl_2_). Protein lysates, 900 μl of 1 μg/μl, were precleared with glutathione sepharose for 1 h at room temperature. The cleared lysates were incubated with GST-rhotekin-sepharose conjugate at 4°C for 30 min. The sepharose conjugate was collected by centrifugation, resuspended in Laemelli buffer, separated by SDS-PAGE, transferred to nitrocellulose and probed with a RhoC antibody developed by our laboratory [55]. Densitometry was performed as described above. The results of triplicate assays were combined and described as percent of Rho activation detected in the wild-type cell line.

### Random Migration and Invasion Assays

Colloidal gold random migration assays were performed as previously described [54]. Random migration assays were also performed using the blue fluorescent bead motility HitKit (Cellomics, Pittsburg, PA). Cells, 200/well in serum free medium (SFM), were seeded onto a lawn of blue fluorescent beads in a BSA coated 96-well plate (BD Biosciences, San Diego, CA). Cells were stimulated 1 h later with 10% FBS, incubated 6 h at 37°C, and fixed. Cells were visualized by fluorescent microscopy, digitally recorded and the areas of phagokinetic tracks measured using the ImageJ software. Migration assays were performed at least three separate times and at least 250 cell tracks were measured per condition in each analysis.

The Matrigel invasion assays were performed as previously described using pre-coated Transwell filters with 8 μ pores [58,54]. Cells were harvested and resuspended in serum-free medium containing 0.1% BSA at a concentration of 3.75 × 10^5 ^cells/ml, and 0.5 ml was added to the top chambers and placed for 24 h at 37°C in a 10% CO_2 _incubator. The cell suspension was aspirated from the top chamber of all three assays. Excess Matrigel was removed from the invasion assay filter using a cotton swab. The filters were then cut away from the Transwell assembly, fixed top side down with methanol to a glass microscope slide, stained with H&E, and the entire surface of the filters counted. The number of cells that moved in serum-free (i.e. no chemoattractant) controls was considered background and was subtracted from the number of cells counted in the samples containing chemoattractant.

### Statistical Analysis

All experiments and assays were performed a minimum of three separate times. Statistical analysis was performed by the University of Michigan Biostatistics Core using the Wilcoxon rank-sum analysis and Students t-test.

## Abbreviations

PC, pancreatic cancer; cav-1, caveolin-1; MAPK, mitogen activated protein kinase; SFM, serum-free medium; GAPDH, glyceraldehyde-3-phosphate dehydrogenase; HPDE, human pancreatic ductal epithelial cells; FBS, fetal bovine serum; RT-PCR, reverse transcriptase polymerase chain reaction; GFP, green fluorescent protein; MβCD, methyl β cyclodextrin.

## Authors' contributions

ML carried out the majority of Western blots, motility and Rho activation assays. MMD carried out RT-PCR and Western blot analysis. SDM contributed to initial experimental design and molecular analysis. MB performed caveolin-1 immunoprecipitation experiments and aided in experimental design. KvG conceived the project, designed the experiments, performed molecular analysis and significantly contributed to the writing of the manuscript.
